# The Morphological Landscape of Bone Marrow Findings in Visceral Leishmaniasis

**DOI:** 10.7759/cureus.104000

**Published:** 2026-02-20

**Authors:** Damleen Mangat, Anshu Palta, Anita Tahlan, Sanjay D'Cruz

**Affiliations:** 1 Pathology, Government Medical College and Hospital, Chandigarh, Chandigarh, IND; 2 General Medicine, Government Medical College and Hospital, Chandigarh, Chandigarh, IND

**Keywords:** bone marrow, cytology, hematopathology, histopathology, leishmaniasis, morphology, parasite density

## Abstract

Background and aim

Visceral leishmaniasis (VL) remains a major parasitic disease globally, with a high burden in the Indian subcontinent. Diagnosis relies on identifying *Leishmania donovani* amastigotes in bone marrow aspirates and biopsies; however, detailed characterization of associated marrow morphology is limited in the current literature. This study aimed to analyze bone marrow aspirate and trephine biopsy findings in visceral leishmaniasis and assess correlations between parasite load and key hematological parameters.

Methods

This retrospective study included 42 confirmed VL cases diagnosed between January 2003 and March 2025 at a tertiary center hospital in India. Clinical data, peripheral blood films, bone marrow aspirates (Romanowsky stain), and trephine biopsies (hematoxylin-eosin stain) were reviewed. Average parasite density (APD) was quantified using standardized oil immersion counts. Morphological features, including cellularity, plasmacytosis, hemophagocytosis, and stromal changes, were recorded. Statistical associations between APD and clinical or hematological variables were assessed using chi-square and Fisher’s exact tests.

Results

The cohort (M:F=2.8:1; aged 5-55 years) universally presented with fever (n=42; 100%), with splenomegaly (n=37; 88.1%) and hepatomegaly (n=33; 78.6%) commonly observed. Pancytopenia was observed in 73.8% (n=31) of cases. Bone marrow findings included hypercellularity (n=34; 80.95%), plasmacytosis (n=37; 88.0%), increased histiocytes (n=34; 80.9%), and hemophagocytosis (n=22; 52.38%). APD distribution was as follows: 1+ (n=6; 14.29%), 2+ (n=16; 38.10%), 3+ (n=13; 30.95%), and 4-5+ (n=7; 16.6%). Higher parasite load correlated significantly with splenomegaly (p=0.031), pancytopenia (p=0.018), and plasmacytosis (p=0.024). Additional findings included dyserythropoiesis (n=16; 38.1%), increased eosinophilic precursors (n=18; 42.86%), myelofibrosis (n=6; 14.2%), and a rare case of gelatinous transformation.

Conclusion

Combined assessment of aspirate cytology and trephine histology enhances diagnostic sensitivity in VL. Recognition of characteristic marrow alterations supports timely diagnosis and reduces unnecessary investigations, particularly in non-endemic settings.

## Introduction

Visceral leishmaniasis (VL), known as kala azar, "black fever," represents one of the most significant parasitic diseases impacting global public health [1]. This chronic infectious disease is caused by the obligate intramacrophagal protozoan parasite *Leishmania donovani*, transmitted via the bite of infected sandflies (Phlebotomus species) [2]. The disease predominates in tropical/subtropical regions, with over 90% of global cases on the Indian subcontinent [3].

Clinically, VL presents as a chronic illness characterized by irregular fever patterns, progressive emaciation, generalized weakness, marked hepatosplenomegaly, pancytopenia, and hypergammaglobulinemia [4-6]. Without treatment, fatality exceeds 90% [7,8].

Diagnostic challenges arise from a non-specific presentation mimicking malaria, tropical splenomegaly, disseminated tuberculosis, malnutrition, and hematologic malignancies [9-11]. Definitive diagnosis requires microscopic demonstration of 2-4 μm round, aflagellate amastigotes (Leishman-Donovan bodies) with a distinctive nucleus and kinetoplast in bone marrow, spleen, or liver aspirates [12-15].

While bone marrow aspirate smears are established for parasite detection, comprehensive trephine biopsy descriptions remain limited, particularly vital in non-endemic areas [16,17]. This study evaluates morphological findings, quantifies parasite load using standardized grading, and correlates parasitemia with hematological parameters.

## Materials and methods

A retrospective study was conducted in the hematology section of the Department of Pathology, Government Medical College and Hospital, Sector 32, Chandigarh, from January 2003 to March 2025, i.e., over a period of 22 years. Bone marrow aspiration was performed bilaterally from the posterior superior iliac bone with a disposable bone marrow needle under 1% lignocaine (local anesthesia).

Inclusion and exclusion criteria

Forty-two cases diagnosed as visceral leishmaniasis were included in the study. The cases in which all the parameters of the current study were not available were excluded from the study. The detailed clinical data and slides of peripheral blood film (PBF), bone marrow aspirate (May-Grünwald Giemsa {MGG}-stained), and trephine biopsies (H&E-stained), performed in all cases, were retrieved from archival material. The morphological findings observed on bone marrow aspirate cytology and trephine biopsy were reviewed. Counting of Leishman-Donovan (LD) bodies was done on bone marrow aspirates under oil immersion.

Parasite density quantification

LD bodies were counted in 100 oil-immersion high-power fields (1000×). Average parasite density classified into the following using Chulay-Bryceson system: grade 1+ (1-10 LD bodies/1,000 fields), grade 2+ (1-10 LD bodies/100 fields), grade 3+ (1-10 LD bodies/10 fields), grade 4+ (1-10 LD bodies/field), and grade 5+ (10-100 LD bodies/field) [18].

Morphological assessment

Hemophagocytosis was graded as follows: 0 (absent), 1+ (<2 histiocytes/slide), 2+ (2-5), 3+ (>5) histiocytes with hemophagocytosed material per slide. Aspirates assessed cellularity, myelodysplasia, eosinophilia, percentage of plasma cells, and histiocytosis and iron stores. Trephines evaluated cellularity, fibrosis, granulomas, lymphoid aggregates, and gelatinous transformation.

Statistical analysis

Chi-square/Fisher’s exact tests were used to assess correlations between parasite load (1-2+ vs. 3-5+). Associations were considered statistically significant at p<0.05. The statistical analysis was performed using SPSS v26.0 (Armonk, NY: IBM Corp.).

## Results

Patients' ages ranged from 5 to 55 years (71.4% were <30 years). Out of these, males comprised 73.8% (n=31) (M:F=2.8:1). Fever (n=42; 100%), splenomegaly (n=37; 88.1%), hepatomegaly (n=33; 78.6%), and lymphadenopathy (n=4; 9.5%) were observed. Pancytopenia (n=31; 73.8%) and bicytopenia (n=8; 19.0%) were noted. Demographic and clinical details of the study are illustrated in Table 1.

**Table 1:** Patient demographics and clinical presentation (n=42).

Variables	n	%
Age distribution (years)		
5-15	12	28.6
16-30	18	42.9
31-45	8	19.0
>45	4	9.5
Gender		
Male	31	73.8
Female	11	26.2
Clinical presentation		
Fever	42	100
Splenomegaly	37	88.1
Hepatomegaly	33	78.6
Lymphadenopathy	4	9.5
Serology		
Aldehyde test positive	20	47.6

Detailed marrow aspirate findings showed hypercellularity in 34 cases (80.95%), myelodysplasia in 21 (50.0%), dyserythropoiesis in 16 (38.1%), dysgranulopoiesis in 14 (33.3%), and dysmegakaryopoiesis seen in six cases (14.3%). Further eosinophilia (n=18; 42.86%), plasmacytosis (n=31; 88.0%), and histiocytosis (n=34; 80.95%) were also documented in these cases. Hemophagocytosis was observed in 22 cases (52.38%), with 12 cases (54.55%) having grade 1+ hemophagocytosis. Iron stores of >3+ were seen in 16 cases (38.1%). LD bodies localized within macrophages were found in 38 cases (90.48%). The bone marrow aspirate findings have been illustrated in Table 2.

**Table 2:** Bone marrow aspirate cytology findings (n=42).

Findings	n	%
Cellularity		
Hypercellular	34	80.95
Normocellular	7	16.67
Hypocellular	1	2.38
Myelodysplasia	21	50.0
Dyserythropoiesis	16	38.1
Dysgranulopoiesis	14	33.3
Dysmegakaryopoiesis	6	14.3
Eosinophilic precursors	18	42.9
Plasma cells (increased)	37	88.0
Histiocytes (increased)	34	80.95
Hemophagocytosis	22	52.4
Iron stores (>3+)	16	38.1

**Figure 1:**
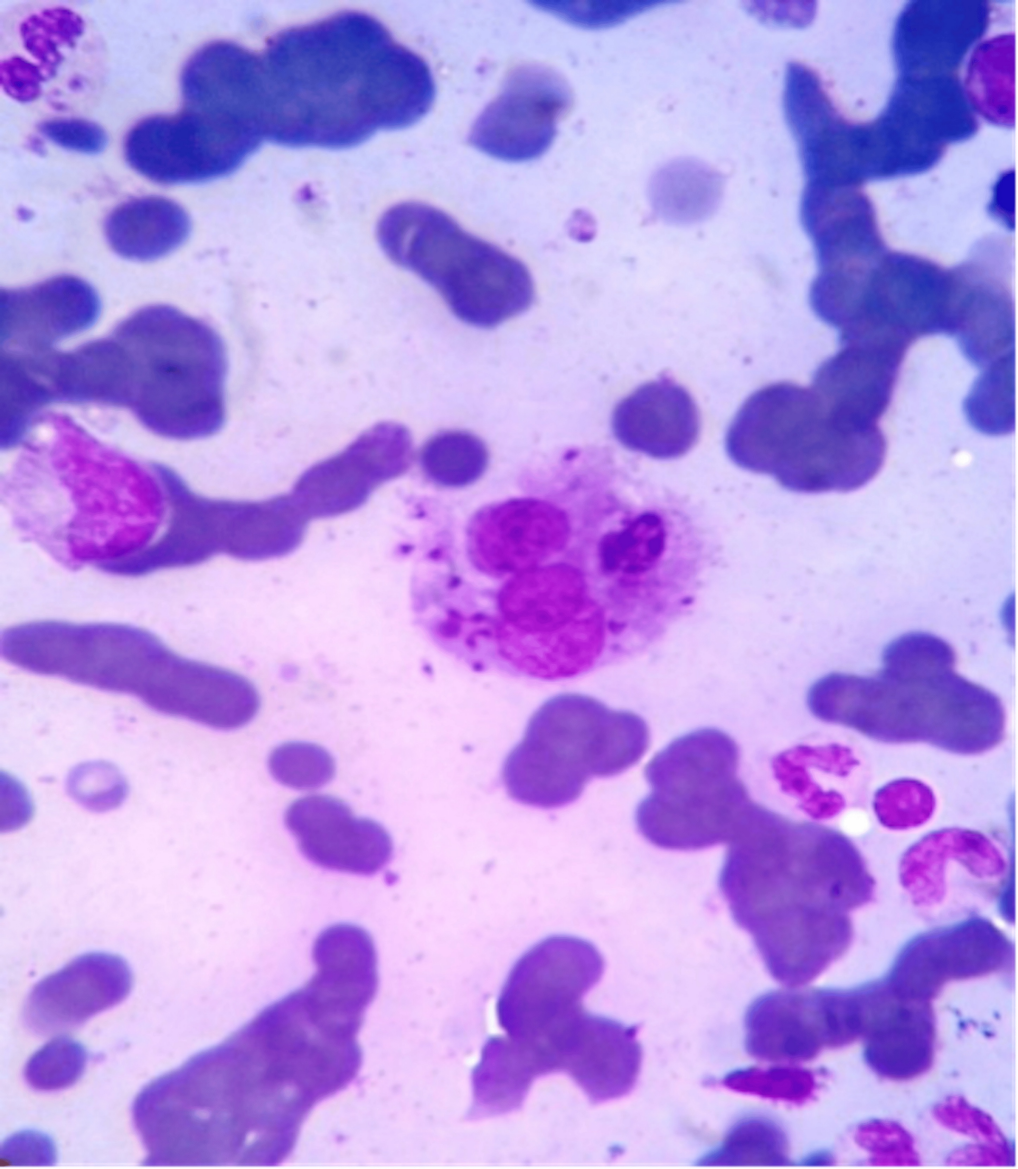
Bone marrow aspirate showing hemophagocytosis (MGG stain, 1000x). MGG: May-Grünwald Giemsa

**Figure 2:**
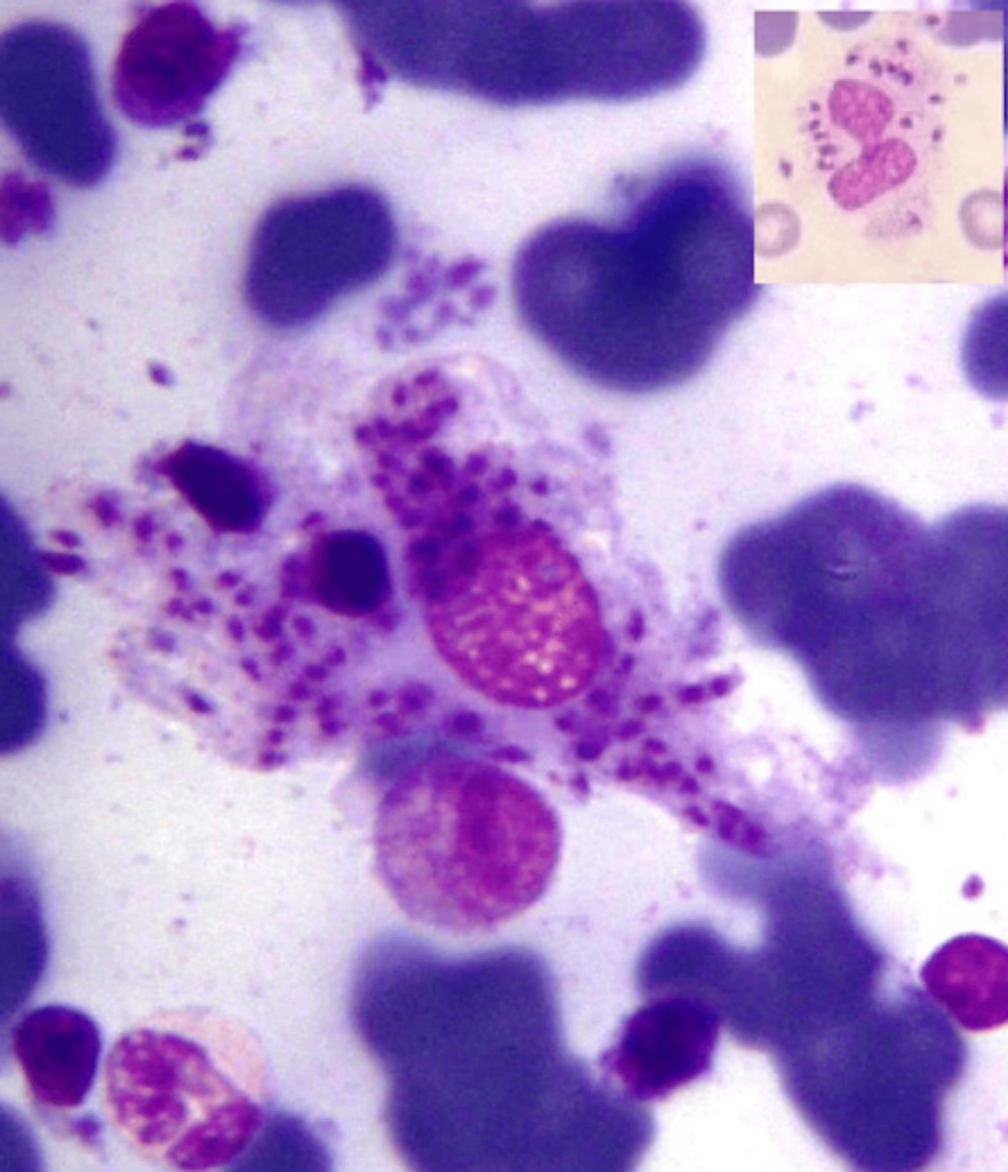
Bone marrow aspirate showing intrahistiocytic LD bodies - APD 5+. Inset shows neutrophil with intracellular LD bodies (MGG stain, 1000x). LD: Leishman-Donovan; APD: average parasite density; MGG: May-Grünwald Giemsa

The intercellular LD bodies in oil immersion are shown in Figures 1, 2. Average parasite density (APD) of grade 2+ was seen in 16 cases (38.10%), 3+ in 13 cases (30.95%), 1+ in six cases (14.29%), 4+ in four cases (9.52%), and 5+ in three cases (7.14%). Histological examination of bone marrow biopsy revealed hypercellularity (n=34; 80.95%), myelofibrosis (n=6; 14.29%), granulomas (n=3; 7.14%), and single cases showed gelatinous transformation (2.38%). APD showed significant associations with splenomegaly (χ²=4.67; p=0.031), pancytopenia (χ²=5.57; p=0.018), and plasmacytosis (χ²=5.12; p=0.024) (Table 3).

**Table 3:** Average parasite density (LD bodies) distribution (n=42). LD: Leishman-Donovan

Grades	Description	n	%
1+	1-10 LD bodies/1,000 fields	6	14.29
2+	1-10 LD bodies/100 fields	16	38.10
3+	1-10 LD bodies/10 fields	13	30.95
4+	1-10 LD bodies/field	4	9.52
5+	10-100 LD bodies/field	3	7.14
6+	>100 LD bodies/field	0	0

## Discussion

VL, caused by *Leishmania donovani*, is transmitted through the bite of infected female sandflies (*Phlebotomus argentipes*) [1]. The disease exhibits endemic distribution in tropical and subtropical regions worldwide, with particular concentration in the Indian subcontinent [19]. According to World Health Organization estimates, leishmaniasis affects approximately two million individuals annually, with visceral disease accounting for roughly 500,000 cases [20]. The global prevalence is estimated at 12 million infected individuals, with approximately 350 million people exposed to infection risk [20]. More than 90% of the world's visceral leishmaniasis cases originate from the Indian subcontinent, rendering this disease a significant public health priority in endemic regions [21].

Diagnosis of visceral leishmaniasis relies on demonstrating the parasite by microscopic examination of specimens obtained by bone marrow aspiration or, less commonly, splenic aspiration [13]. Various cytological and histological features associated with visceral leishmaniasis have been individually described in the published literature. However, comprehensive descriptions of the complete morphological spectrum of visceral leishmaniasis in bone marrow aspiration and biopsy specimens remain surprisingly scarce in contemporary English-language literature. Given the enormous disease burden in endemic regions like India, systematic study of bone marrow examination findings is essential to guide pathologists toward probable diagnosis when clinical suspicion is otherwise low [1,16].

Our study cohort of 42 patients ranged in age from five to 55 years, with the majority of cases (n=30; 71.4%) concentrated in individuals younger than 30 years. This age distribution is consistent with previous published series by Agrawal et al. and Uzair et al. [22,23]. We observed a male-to-female ratio of 2.8:1, reflecting the lack of inherent gender predisposition for visceral leishmaniasis [24].

The clinical presentation in our series demonstrated characteristic features. Fever, present in all 42 patients (100%), is the hallmark symptom of visceral leishmaniasis and typically exhibits an irregular pattern of daily spiking temperatures. This finding aligns with published reports indicating a fever incidence of 98.5% in other series [25]. Splenomegaly was documented in 88.1% (n=37) of our patients, and hepatomegaly was observed in 78.6% (n=33) of cases, comparable to the 83% hepatosplenomegaly incidence reported by Rai et al. [25]. Lymphadenopathy was encountered in only 9.5% (n=4) of our cohort.

Pancytopenia, defined as a simultaneous reduction in hemoglobin concentration, white blood cell count, and platelet count, was observed in 73.8% (n=31) of our cases. This finding closely corresponds to the 75% incidence reported in a case series by Bhatia et al. and slightly exceeds the 96.2% rate in the case series by Chandra et al., which may reflect differences in case selection or laboratory thresholds [26,27]. The pathophysiology underlying pancytopenia in visceral leishmaniasis is multifactorial, involving hypersplenism with enhanced sequestration of blood elements, hemolysis, plasma volume expansion, ineffective erythropoiesis, and aberrant retention of iron by macrophages due to reticuloendothelial system hyperplasia [28].

Hypercellular bone marrow, present in 80.95% (n=34) of our cases, is a characteristic finding in visceral leishmaniasis despite peripheral cytopenias. This apparent paradox, hypercellular marrow with pancytopenia, is well-documented and reflects the pathophysiology of the disease. Thirty-four (80.95%) cases in this study demonstrated hypercellularity, compared with 77.7% in the series by Dhingra et al. and 26% in the series by Chandra et al. [1,27]. The discrepancy likely reflects differences in the criteria for assessing cellularity across patient age groups. Multiple proposed mechanisms explain the emergence of hypercellular marrow in the face of peripheral cytopenias such as (1) splenic sequestration of circulating blood elements, (2) ineffective hematopoiesis with intramedullary destruction of developing cells, (3) hemophagocytosis by activated macrophages, and (4) selective enhancement of myelopoiesis mediated by *Leishmania donovani *infection of marrow stromal macrophages, leading to overproduction of granulocyte-macrophage colony-stimulating factor (GM-CSF) and tumor necrosis factor-alpha (TNF-α) [29,30]. Clinical observations indicate that patients with hypercellular marrow demonstrate superior therapeutic response to antimicrobial therapy [31].

Myelodysplastic changes were identified in 50% (n=21) of our cases. Dyserythropoiesis, manifested as nuclear abnormalities including karyorrhexis (nuclear fragmentation), binucleation, and multinucleation, was documented in 38% (n=16) of cases. The severity grading revealed predominantly severe dyserythropoiesis (n=12; 56.25% of dysplastic cases), which aligns with previously reported data [5]. The mechanism underlying these nuclear abnormalities remains incompletely understood but likely reflects the toxic effects of parasitic burden on developing erythroid precursors combined with ineffective erythropoiesis. These findings, while non-specific, provide additional morphological clues suggesting the diagnosis in clinically unsuspected cases.

Dysgranulopoiesis, including pseudo-Pelger-Huet anomaly (abnormal nuclear lobulation), was observed in 33.3% (n=14) of cases, with pseudo-Pelger-Huet anomaly specifically identified in 11.9% (n=2) of cases. This contrasts with the 25% incidence reported by Bhatia et al. [26]. Such variations likely reflect differences in grading criteria and observer experience.

Dysmegakaryopoiesis, characterized by abnormal megakaryocytic maturation, including hypolobated and micromegakaryocytes, was identified in 14.3% (n=6) of our cases. According to an extensive literature review, this finding has not been systematically reported in the English-language literature on visceral leishmaniasis, and its etiopathology requires extensive research.

Increased plasma cells (plasmacytosis) were observed in 88.0% (n=31) of our cases, an incidence comparable to the 86.7% reported by Dhingra et al. and higher than the 56% reported by Bhatia et al. [1,26]. The mechanism underlying plasma cell accumulation in visceral leishmaniasis involves sustained antigenic stimulation by parasitic antigens, which drive differentiation of B lymphocytes into antibody-producing plasma cells. This marked elevation of plasma cells provides a valuable morphological clue toward suspecting visceral leishmaniasis in unexplained cases.

Increased eosinophilic precursors (eosinophilia) were noted in 42.86% (n=18) of our cases, representing an unusual finding not commonly emphasized in the visceral leishmaniasis literature. Dhingra et al. reported similar findings in 16.66% of their cases; however, no secondary causes of eosinophilia were identified in our cohort [1]. The phenomenon merits further investigation, as the mechanism underlying eosinophilic myelopoiesis in visceral leishmaniasis remains unclear. Potential mechanisms may include parasitic antigenic stimulation or an altered cytokine milieu that promotes eosinophilic differentiation.

Increased histiocytes (morphologically benign but functionally activated macrophages) were observed in 80.95% (n=34) of our cases. This finding reflects the reticuloendothelial system hyperplasia characteristic of visceral leishmaniasis. Hemophagocytosis, defined as phagocytosis of blood cellular elements by histiocytes, was documented in 52.38% (n=22) of our cases. The distribution of hemophagocytosis severity in our series differed from comparison studies.

The present study documented that mild hemophagocytosis (grade 1+) predominated (n=12; 54.55%) in hemophagocytic cases, contrasting with the moderate-grade predominance (32%) and severe cases (12%) reported by Bhatia et al. [26]. These variations likely reflect differences in grading stringency and case selection. The mechanism of hemophagocytosis in visceral leishmaniasis involves activated macrophages responding to parasitic antigenic stimulation combined with abnormal erythropoiesis, generating dysplastic and fragile erythroid precursors susceptible to ingestion.

Increased bone marrow iron stores (grade >3+) were documented in 38.1% (n=16) of our cases, closely paralleling the 33.33% reported by Dhingra et al. [1]. Both increased and decreased iron stores have been variably reported in the literature on visceral leishmaniasis. The mechanism of iron accumulation involves cytokine overproduction (particularly TNF-α), which drives the pathophysiology of anemia of chronic disease through hepcidin upregulation and iron sequestration [30]. Additionally, dyserythropoiesis with destruction of erythroid precursors prevents iron reutilization, leading to net accumulation in reticuloendothelial stores. Conversely, some studies report low iron stores attributable to the poor nutritional status of patients from lower socioeconomic backgrounds in endemic regions [1].

Granulomas, while characteristic of some parasitic and infectious diseases, were identified in only 7.14% of our cases (three of 42 cases). This low incidence contrasts strikingly with the 66% reported by Bhatia et al. [26], but aligns more closely with the 22.22% and 23% reported by Dhingra et al. and Daneshbod et al., respectively [1,32]. This discordance likely reflects diagnosis at earlier disease stages and variable parasite burdens across different endemic regions. Additionally, reduced parasite density may diminish granulomatous inflammation as a host response mechanism.

Gelatinous transformation, a rare finding characterized by fat cell atrophy, focal loss of hematopoietic cellularity, and accumulation of extracellular eosinophilic amorphous material rich in hyaluronic acid, was identified in a single case (2.38%) in our cohort. This finding has been reported in the English-language literature on only two previous occasions, by Dhingra et al. (5.6% incidence) and Varma et al. (8.1% incidence), making this only the third systematic report of this phenomenon in association with visceral leishmaniasis [1,33]. Gelatinous transformation is also recognized in patients with anorexia nervosa, acute febrile states, and advanced HIV/AIDS [33]. Whether gelatinous transformation in visceral leishmaniasis represents a direct parasitic effect or a manifestation of the systemic inflammatory response to acute infection remains an open question requiring further investigation.

Quantification of LD body burden using the standardized Chulay-Bryceson grading system revealed predominantly moderate parasite densities. The mode parasite density grade was grade 2+ (1-10 LD bodies per 100 fields), observed in 38.10% (n=16) of cases. Combined grades 1-3+ accounted for 83.33% (n=35) of cases, indicating that most patients had moderate-to-low parasite burdens at the time of diagnosis. Higher parasite grades (4-6+) represented only 16.67% (n=7) of the cohort, with no cases achieving the maximal grade 6+ (>100 LD bodies per field).

Statistical analysis revealed significant associations between parasite load and three clinical/hematological parameters of splenomegaly, pancytopenia, and plasmacytosis. Splenomegaly (χ²=4.67; p=0.031) was more prevalent and correlated significantly with elevated parasite density, likely reflecting direct parasitic infiltration and macrophage activation within the splenic red pulp. Pancytopenia (χ²=5.57; p=0.018) was severe and showed a significant association with increased parasitemia, consistent with the hypothesis that higher parasite burden intensifies hypersplenism and reticuloendothelial macrophage activation. Elevated plasma cell percentages (plasmacytosis) correlated significantly with increased parasite density (χ²=5.12; p=0.024).

Notably, hemophagocytosis and other bone marrow features showed trends toward association with increased parasite load but did not achieve statistical significance, likely reflecting the limited sample size. Other published investigations by Marwaha et al. and Hamid and Gobah have reported similar correlations between parasite burden and hematological parameters, though some variations exist [34,35].

Leishman-Donovan bodies were identified intracellularly within macrophages in 90.48% (n=38) of cases, representing the characteristic localization. Strikingly, intracellular organisms were identified within neutrophils in only one case (2.38%), suggesting that while macrophages represent the primary parasitic habitat, rare infections of other myeloid lineages may occur. This observation corroborates the obligate intramacrophagal nature of *L. donovani* while acknowledging rare exceptions. Table 4 presents comparative data from our study alongside previously published series by Agrawal et al., Bhatia et al., and Chandra et al. [22,26,27].

**Table 4:** Comparison of findings with previous published series.

Findings	Bhatia et al. [26] (%)	Chandra et al. [27] (%)	Agrawal et al. [22] (%)	Present study (%)
Pancytopenia	75.0	96.2	25.0	73.8
Monocytosis	62.0	18.5	-	38.1
Hypercellularity	-	26.0	5.0	80.95
Plasmacytosis	56.0	96.2	60.0	88.0
Increased histiocytes	81.0	100.0	-	80.95
Hemophagocytosis	75.0	70.3	-	52.4
Dyserythropoiesis	38.0	-	40.0	38.1
Eosinophilia	-	14.8	-	42.9
Granulomas	66.0	5.2	-	7.1
Fibrosis	19.0	10.4	-	14.3
Gelatinous transformation	-	-	-	2.3

Our findings demonstrate general concordance with published reports regarding pancytopenia, plasmacytosis, histiocyte elevation, and hemophagocytosis. Notable variations exist regarding granuloma formation (lower in our series) and eosinophilia (higher in our series), likely reflecting case selection, endemic region differences, and diagnostic criteria variations.

Limitations

The sample size of the 42-case cohort for this substantially rare disease limits statistical power for multivariate analysis and subgroup stratification. Additionally, the single-center cohort results, derived from a single tertiary medical center, may not represent all geographic regions or institutional practices.

Clinical implications

The comprehensive morphological assessment combining bone marrow aspirate cytology with trephine histology substantially enhances diagnostic sensitivity and specificity for visceral leishmaniasis. Recognition of the characteristic constellation of findings, including parasite demonstration, hemophagocytosis, prominent plasmacytosis, myelodysplastic features, and increased histiocytes, facilitates early and accurate diagnosis, particularly in non-endemic regions where clinical suspicion is often limited.

These findings reduce reliance on advanced diagnostic modalities (including serological enzyme-linked immunosorbent assay (ELISA), molecular PCR testing, and imaging) and unnecessary clinical workup, thereby reducing healthcare costs and expediting initiation of appropriate anti-leishmanial therapy. Furthermore, quantification of parasite load may provide prognostic information, as preliminary evidence suggests parasite density correlates with disease severity and potentially with therapeutic response.

## Conclusions

Visceral leishmaniasis presents a diagnostic challenge due to the non-specific nature of its clinical and laboratory manifestations. This comprehensive 22-year retrospective study of 42 cases demonstrates a characteristic and reproducible spectrum of morphological findings on bone marrow aspirate cytology and trephine histology. The combination of features, including systematic parasite demonstration, hemophagocytosis, marked plasmacytosis, myelodysplastic features, and reticuloendothelial hyperplasia, provides pathologists with morphological clues essential for clinching diagnosis, particularly in non-endemic regions where clinical suspicion is low.

Familiarity with these findings and appropriate and vigilant microscopic examination facilitates early and accurate diagnosis of this potentially fatal parasitic infection, enabling timely initiation of anti-leishmanial therapy. Furthermore, systematic quantification of parasite load using standardized grading provides additional prognostic and therapeutic information. The association of parasite burden with splenomegaly, pancytopenia, and plasmacytosis offers insights into disease pathophysiology and may guide clinical management decisions. Future investigations incorporating larger cohorts, molecular confirmation of *Leishmania donovani* species identification, and longitudinal therapeutic response data would further elucidate the clinicopathological spectrum and prognostic significance of bone marrow findings in visceral leishmaniasis.
